# Bacterial etiologic agents causing neonatal sepsis and associated risk factors in Gondar, Northwest Ethiopia

**DOI:** 10.1186/s12887-017-0892-y

**Published:** 2017-06-06

**Authors:** Tsehaynesh G/eyesus, Feleke Moges, Setegn Eshetie, Biruk Yeshitela, Ebba Abate

**Affiliations:** 1 Amhara Regional Health Bureau, South Gondar Zonal Health Bureau, Debre Tabor, Ethiopia; 2 Department of Medical Microbiology, School of Biomedical and Laboratory Sciences, College of Medicine and Health Sciences, University of Gondar, Gondar, P. O. Box: 196, , Ethiopia; 3 Armauer Hansen Research Institute (AHRI), Addis Ababa, Ethiopia

**Keywords:** Neonate, Sepsis, Antimicrobial susceptibility, Gondar, Ethiopia

## Abstract

**Background:**

Neonatal sepsis is a blood stream infection which is seen in the first month of life of the neonate. Bacterial profile of neonatal septicemia is constantly changing thus, current knowledge on the patterns of bacterial isolates, its antibiotic resistance profile, and associated factors, are essential to design and implement appropriate interventions. Therefore**,** the aim of this study was to identify bacterial etiologic agents, their antimicrobial susceptibility pattern and associated risk factors of neonatal sepsis among neonates.

**Methods:**

A cross- sectional study was conducted among neonates suspected to sepsis attending University of Gondar Hospital from September/2015 to May/2016. A total of 251 consecutive neonates with clinical sign and symptoms of sepsis were included in the study. Blood sample was collected and directly inoculated into Trypton soya broth bottle and incubated at 37 °C. After 24 h of incubation it was sub- cultured in to blood agar plate, chocolate agar plate, manitol salt agar and Macconkey. The bacterial pathogens and antimicrobial susceptibility tests were identified using standard microbiological methods. Bivariate and multivariate logistic regressions were used to identify possible associated risk factors. Prior to the study ethical clearance was obtained from the School of Biomedical and Laboratory Sciences, University of Gondar.

**Results:**

Of the 251 study participants suspected of neonatal sepsis, 117 (46.6%) showed bacterial growths, of them 120 bacteria were isolated. Gram positive bacteria were commonly isolated 81 (67.5%).The commonly isolated bacterial species were *S. aureus* 49 (40.8%) followed by coagulase negative *Staphylococci* 26 (21.6%) and *K. pneumoniae*19 (15.8%). The overall rate of multidrug resistance isolates was 78 (65%: CI 95%: 56.7–72.5%). Multidrug resistant (MDR) among Gram positive and negative bacteria were 56 (69.1%) and 22 (56.4%), respectively. Independent risk factors for the occurrence of neonatal sepsis were; Apgar score < 7/5 min (Adjusted odds ratio [AOR] =0.5), birth weight < 1.5 kg (AOR = 12.37), birth weight, 1.5–2.5 kg (AOR = 2.6), gestational week <37 weeks (AOR = 9) and caesarian section delivery (AOR = 5.2).

**Conclusion:**

The isolation rate of bacterial pathogens in neonatal sepsis was considerably high. In addition, nearly 70% of isolates were MDR strains. Low birth weight, low Apgar score, preterm delivery and caesarian section modes of delivery were associated risk factors. Therefore, appropriate antenatal care follow up, and health education should be encouraged, especially on the importance of natural way of delivery.

**Electronic supplementary material:**

The online version of this article (doi:10.1186/s12887-017-0892-y) contains supplementary material, which is available to authorized users.

## Background

Neonatal sepsis is a systemic infections of the newborn such as septicemia, meningitis, pneumonia, arthritis, osteomyelitis, and urinary tract infections [1]. The clinical syndrome of the disease can be influenced by the virulence of the pathogen, the portal of entry, the susceptibility and response of the host, and the temporal evolution of the condition [2]. Fever, temperature instability, vomiting, diarrhea, irritability, lethargy, breathing problem, low blood sugar, jaundice, reduced sucking and seizures are the most common symptoms of neonatal sepsis [3]. Neonatal sepsis may be classified according to the time of onset of the disease: early onset and late onset. Early-onset of neonatal sepsis refers to the presence of a confirmed infection in the blood or cerebrospinal fluid (CSF) of patients younger than 3 days of life, and late-onset of neonatal sepsis refers to the onset of such infection between 3 and 28 days [4, 5].

The distinction has clinical relevance, as early onset of neonatal sepsis disease is mainly occurred before and during delivery, whereas late onset of neonatal sepsis is possible due to bacteria acquired after delivery through hospital or community acquired source of infections [6, 7]. Organisms causing early onset of neonatal sepsis are typically colonizers of the maternal genitourinary tract, leading to contamination of the amniotic fluid, placenta, cervix, or vaginal canal. The pathogen may ascend when the amniotic membranes rupture or prior to the onset of labor, causing an intra-amniotic infection [8].

Bacteria, such as *Streptococcus*, *L. monocytogenes, E. faecalis,E. faecium*, group D *Streptococci*, α-hemolytic *Streptococci* and *Staphylococci,S. pneumoniae,H. influenzae* type B, are recognized as the principal cause of early neonatal sepsis. Less commonly, *N. meningitidis* and *N. gonorrhoeae* have been also reported as a cause of neonatal septicemia and Gram-negative enteric organisms predominantly *E. coli*, *Klebsiella* species are included [9, 10]. On the other hand, late-onset sepsis is predominantly caused by *Staphylococci* species and *E. coli* and most frequently related with low birth weight of infants and use of intravascular catheters, endo-tracheal intubation, assisted ventilation, surgery, contact with hand of colonized personnel and contact with contaminated equipment as the main risk factors for late onset of neonatal sepsis. Sometimes *E. cloacae* or *Citrobactor* from blood or CSF may be due to contaminated feedings. Contaminated respiratory equipment is suspected in outbreaks of hospital-acquired *P. aeroginosa* pneumonia or sepsis [11, 12].

Neonatal sepsis remains public health threat and contributing a significant challenge to the management of care groups for neonates and infants. It has been explained that neonates are at the highest risk for bacterial sepsis, with the prevalence at 1 to 10 per 1000 live births worldwide [13]. Globally each year over 4 million neonates died within 28 days of birth [14]. Neonatal mortality is still the highest mortality in human life. It has a close relation to infant mortality rate, which is used as the indicator to assess health development in countries [15]. Besides, a recent global report has also claimed that the infant mortality rate was 29 per 1000 live births [16]. The most common causes of death in the neonatal period are infections, including septicemia, meningitis, respiratory infections, diarrhea, and neonatal tetanus (32%), followed by birth asphyxia and injuries (29%), and prematurity (24%) [17].

Aside from the burden, continuous evolution of drug resistance of pathogens causing neonatal sepsis becomes a potential disastrous problem. The situation is worse in developing countries because of lack of legislation, over-the-counter sale of antibiotics and poor sanitary conditions. Notably, methicillin resistance *S.aureus* (MRSA), extended spectrum beta lactamase (ESBL) producing bacteria and MDR Gram negative organisms represent the principal setbacks to fight against infections. Most Gram negative bacteria are now resistant to ampicillin and cloxacillin and many are becoming resistant to gentamicin [18, 19].

The pattern of bacterial organisms causing neonatal sepsis constantly changes and the emergence of resistant bacteria has complicated the problem further. Therefore, this study aimed to assess bacterial etiologic agents, their antimicrobial susceptibility pattern and possible associated risk factors among neonatal patients with sepsis.

## Methods

### Study area, study design and study population

A prospective cross-sectional study was conducted among all admitted neonates for sepsis at the University of Gondar Hospital from September/2015 to May/2016. Diagnosis of neonatal sepsis remains challenging in this hospital, and mainly depends on clinical decision.

### Inclusion criteria

Neonates with clinical sign and symptoms of sepsis at the time of admission or who develop sepsis during their stay in hospital were included in this study.

### Exclusion criteria

Neonates with sepsis, who started antibiotic therapy, were excluded from the study.

### Study variables

Presence of bacterial infection was dependent variable, whereas independent variable were age, sex, occupation, weight at birth, gestational week, mode of delivery, maternal health condition (fever, urinary tract infection), bottle feeding, Apgar score at 5 min, and skin or local infection.

### Definition of terms

Bacteremia means the presence of bacteria in the blood stream, which may or may not be caused by infectious agents. Septicemia, on the other hand, it is serious disease characterized by systemic signs, and the presence of bacteria and their toxins in the blood stream.

### Data collection and laboratory methods

#### Socio demographic data

Socio**-** demographic data of the study participants and associated risk factors for neonatal sepsis were collected using structured questionnaire. Questionnaire was checked for its completeness and validity prior to the collection of data. The pre test of the questionnaire was conducted among neonates with suspicion of sepsis in Bahirdar hospital (Additional file 1: Questionnaire).

### Sample collection and processing

After getting written consent from guardian two bottle of blood samples (1 ml for each bottle) from two different sites of peripheral vein were collected aseptically (disinfecting with 70% alcohol and 2% tincture of iodine) by experienced nurses prior to any antibiotic use. The collected blood sample was inoculated directly into Trypton soya broth blood culture medium bottles (Oxoid LTD) and sent to the medical microbiology laboratory for culture and drug susceptibility testing. The blood cultures were incubated aerobically at 37 °C and observed daily for the first 3 days for the presence of visible microbial growth by one of the following: haemolysis, air bubbles (gas production), and coagulation of broth. At the same time, subcultures were made during successive days on enriched and selective media including blood agar plate (Oxoid LTD), chocolate agar plat (incubated at 5% CO_2_ atmosphere), MacConkey (Oxoid Ltd. Basingstoke, Hampshire, UK), and manitol salt agar plates and examined for growth after 24–48 h of incubation.

The same protocol was repeated until the 7th day before blood cultures were considered to be free of microorganisms. Isolates obtained were identified by standard microbiological techniques namely, Gram staining, colony characteristics, and biochemical properties including catalase, coagulase (free and bound), growth on manitol salt agar, and hemolytic activity on blood agar plate for Gram positive isolates, and triple sugar iron, motility, indole, citrate utilization, urease, oxidase and H2S production for Gram negative isolates [20]. As described above two aerobic blood culture bottles were used for each patient, and growth in both bottles was considered as positive. Thus, growth of bacteria only from one bottle did not regard as pathogen and considered as contamination.

### The antimicrobial susceptibility tests

Antimicrobial susceptibility test was carried out for each identified bacteria based on Clinical and Laboratory Standard Institute guideline by using disc diffusion method on Muller Hinton agar for non-fastidious organisms and Muller Hinton agar with 5% defibrinated sterile sheep blood for fastidious organisms [21].

Three to five pure colony of the test organism was taken by using a sterile wire loop and emulsify in 2 ml of nutrient broth. The bacterial suspensions turbidity was matched and checked with 0.5 McFarland standards. Then a sterile cotton swab was dipped in to the suspension and squeeze free from excess fluid against the side of test tube. The test organisms were uniformly seed on the surface of Muller-Hinton agar for non fastidious group and Muller Hinton agar with 5% defibrinated sterile sheep blood for fastidious group and expose to a concentration gradient of antibiotic diffusing from antibiotic impregnated paper disc into the agar medium and the medium was incubated at 35 °C for 18–24 h [22].

The following antimicrobial disks were used (Oxoid UK), Amoxicillin-clavulanate (AMC:20/10 μg), penicillin (P:10 unit), trimethoprim-sulfamethoxazole (SXT:1.25/23.75 μg), erythromycin (E:15 μg), tetracycline (TE: 30 μg), clindamycin (DA:2 μg), chloramphenicol (CAF:30 μg), ampicillin (AMP:10 μg), gentamicin (CN:10 μg), amikacin (AK:30 μg), ciprofloxacin (CIP: 5 μg), ceftazidime (CAZ: 30 μg),ceftriaxone (CRO:30 μg) and cefoxitin (CXT:30 μg). Grades of susceptibility pattern were interpreted by comparison of the zone of inhibition according to Clinical and Laboratory Standard Institute 2014 guideline [21].

### Quality control

All materials, equipment and procedures were adequately controlled. Pre-analytical, analytical and post-analytical stages of quality assurance that are incorporated in standard operating procedures of the medical microbiology laboratory were strictly followed. The sterility of culture media was ensured by incubating 5% of each batch of the prepared media at 37^o^c for 24 h. Performance of all prepared media was also checked by inoculating international standard-strains such as *E.coli* (ATCC 25922) Gram negative bacteria, *S. aureus* (ATCC 25923) for Gram positive bacteria and *N. gonorrhoeae* (ATCC49226) for fastidious bacteria. To standardize the inoculums density of bacterial suspension for the susceptibility test, 0.5 McFarland standards was used [20]. To ensure the accuracy of data, double data entry method was used.

### Data analysis and interpretation

Data were collected and analyzed for the presence and frequency of different bacterial isolates, antibiotic susceptibility pattern and associated risk factors. The findings were entered and analyzed using SPSS version 20. Frequencies and cross tabulations were used to summarize descriptive statistics. Univariate and multivariate logistic regression analyses were used to assess the possible risk factors of neonatal sepsis infection. Descriptive statistics was also used to explain antimicrobial susceptibility patterns. Odds ratio and 95% confidence interval was computed to assess the presence and degree of association between dependent and independent variables. *P*-value <0.05 was considered statistically significant for all cases.

## Results

### Characteristics of study participants

Of the total study participants (*n* = 251), 149 (59.4%) were male, 184 (73.3%) of the neonates were age ≤ 3 days (early onset) and146 (58.2%) were from urban residence. Of the total neonates 46 (18.3%) had very low birth weight (< 1.5 kg), 92 (36.6%) were preterm (<37 week gestation), 160 (63.7%) were delivered by spontaneous vaginal delivery. It was observed that 55 (21.9%) of the neonates mothers had history of urinary tract infection (UTI) during gestational period (Table 1).

**Table 1:** Distributions of bacterial isolates per characteristics of study participants with neonatal septicemia attending the University of Gondar Hospital, North west Ethiopia, September/2015 to May/2016, (*N* = 251)

Characteristic		Organism isolated		
		Yes n (%)	No n (%)	Total n (%)
Age in (day)	0–3	84 (71.8)	100 (74.6)	184 (73.3)
	>3–28	33 (28.2)	34 (25.4)	67 (26.7)
Sex	Male	68 (58.1)	81 (60.4)	149 (59.4)
	Female	49 (41.9)	53 (39.6)	102 (40.6)
Residence	Urban	70 (59.8)	76 (56.7)	146 (58.2)
	Rural	47 (40.2)	58 (43.3)	105 (41.8)
Prolonged labor	No	103 (88)	114 (85.1)	217 (86.5)
	Yes	14 (12)	20 (14.9)	34 (13.5)
Prolonged rupture of membrane	No	94 (80.3)	104 (77.6)	198 (78.9)
	Yes	23 (19.7)	30 (22.4)	53 (21.1)
Fever during per partum period	No	102 (87.2)	117 (87.3)	219 (87.3)
	Yes	15 (12.8)	17 (12.7)	32 (12.7)
Mode of delivery	CS	52 (44.4)	29 (21.6)	81 (32.3)
	SVD	60 (51.3)	100 (74.6)	160 (63.7)
	Instrumental	5 (4.3)	5 (3.7)	10 (4)
History of UTI during gestational period	No	92 (78.6)	104 (77.6)	196 (78.1)
	Yes	25 (21.4)	30 (22.4)	55 (21.9)
Getting treatment for UTI	No	12 (10.3)	15 (11.2)	27 (10.8)
	Yes	13 (11.1)	15 (11.2)	28 (11.2)
Gestational week	Preterm <37 wks	75 (64.1)	17 (12.7)	92 (36.7)
	Term 37–42 wks	42 (35.9)	101 (75.4)	143 (57)
	Post term >42 wks	0 (0)	16 (11.9)	16 (6.4)
Birth Weight	Very low <1.5 kg	40 (34.2)	6 (4.5)	46 (18.3)
	Low 1.5–2.5 kg	37 (31.6)	24 (17.9)	61 (24.3)
	Normal >2.5 kg	40 (34.2)	104 (77.6)	144 (57.4)
Apgar score	Low <7/ 5 min	49 (41.9)	69 (51.5)	118 (47)
	Normal >7 /5 min	68 (58.1)	65 (48.5)	133 (53)
Previous history of neonatal admission	No	109 (93.2)	125 (93.3)	234 (93.2)
	Yes	8 (6.8)	9 (6.7)	17 (6.8)
Bottle feeding	No	112 (95.7)	127 (94.8)	239 (95.2)
	Yes	5 (4.3)	7 (5.2)	12 (4.8)
Skin/Local infection	No	108 (92.3)	126 (94)	234 (93.2)
	Yes	9 (7.7)	8 (6)	17 (6.8)

Key: *CS* Cesarean Section, *SVD* Spontaneous Vaginal Delivery

### Magnitude and bacterial profiles among neonatal septicemia

Of the study participants with neonatal septicemia, 117 (46.62%) showed bacterial growth and of these 120 different kinds of bacterial pathogens were identified. Three of blood cultures (2.5%) showed mixed growth (poly microbial), while the 114 (97.5%) showed non duplicate growth. The rest 134 (53.4%) had no bacterial growth. Gram positive bacterial species were commonly isolated 81 (67.5%) than the Gram negative bacterial species 39 (32.5%). The commonly isolated bacteria were *S. aureus* 49 (40.9%) followed by CoNS 26 (21.7%) and *K. pneumoniae* 19 (15.8%) (Table 2).

**Table 2:** Distribution of bacterial isolates from neonates based on their age of admission at University of Gondar Hospital, Northwest Ethiopia, September/2015 to May/2016

Isolated organism	Age at time of admission		Total isolates N (%)
	0–3 day n (%)	>3–28 day n (%)	
*S. aureus*	34 (69.38)	15 (30.62)	49 (40.8)
Cons	18 (69.2)	8 (30.8)	26 (21.7)
*S. pyogenes*	5 (83.33)	1 (16.67)	6 (5)
*E. coli*	9 (75)	3 (25)	12 (10)
*K. pneumoniae*	13 (68.43)	6 (31.57)	19 (15.8)
Others	08 (100)	0 (0)	08 (6.7)
Total	87 (72.5)	33 (27.5)	120 (100)

Key: Others: *Serratia* (*n* = 1), *E. cloacae* (*n* = 4) and *K. rhinose* (*n* = 3)*,* CoNs: Coagulase Negative *Staphylococcus*

The predominant organisms during the first 3 days of life were Gram positive, accounting for 57 (65.5%) of the 87 (72.5%) isolates. Between 3 and 28 days of life, Gram negative and Gram positive isolates accounted for 9 (27.28%) and 24 (72.72%) of the 33 isolated organisms, respectively. The single predominant Gram positive organism in both age groups was *S. aureus* 49 (40.83%). Among Gram negatives, *K. pneumonia* 19 (15.83%) was the predominant causative agent in both age groups (Table 2).

### Antimicrobial susceptibility pattern of bacterial isolates from neonatal septicemia

From the total isolates only 11 (9.2%, 95% CI, 4.4–15%) isolates were susceptible to all tested antibiotics. Over all in Gram positive isolates high resistance rate were observed to penicillin 42 (51.8%), ampicillin 30 (37%) and trimethoprim- sulfamethoxazole 34 (42%). On the other hand, Gram positive bacteria showed least resistance rate to clindamycin 7 (8.6%) andciprofloxacin 16 (19.7%). As indicated Table 3, species specific antibiotic resistance rates showed that more than 35% of *S. aureus* isolates were resistant to penicillin, ampicillin, Trimethoprim- sulfamethoxazole, gentamicin anderythromycin.

**Table 3:** Antimicrobial susceptibility patterns of Gram positive bacteria from blood cultures in neonatal sepsis, University of Gondar Hospital, Northwest Ethiopia, September/2015 to May/2016

Bacterial isolates	Resistance rate N (%)											
	P	AMP	OXA	CXT	SXT	CIP	CRO	TE	CAF	E	CN	DA
*S. aureus*(*n* = 49)	25 (51)	20 (41)	13 (27)	13 (27)	18 (37)	10 (20)	12 (24.5)	16 (33)	15 (30.6)	18 (37)	19 (38.8)	04 (8.1)
CoNs (*n* = 26)	17 (65)	10 (38)	10 (38)	10 (38)	12 (46)	05 (19)	08 (30.8)	11 (42)	10 (38)	11 (42)	14 (53.8)	02 (7.7)
*S.pyogenes*(*n* = 6)	0 (0)	0 (0)	1 (17)	1 (17)	4 (67)	1 (17)	2 (33.3)	1 (17)	1 (17)	1 (17)	2 (33.3)	1 (17)
Total = 81	42 (52)	30 (37)	24 (30)	24 (30)	34 (42)	16 (20)	22 (27.2)	28 (34)	22 (27)	30 (37)	28 (35)	07 (8.6)

Keys: *CoNS* Coagulase negative *Staphylococcus DA* Clindamycin, *CXT* Cefoxitin, *E* Erythromycin, *OXA* Oxacillin, *P* penicillin, *SXT* Trimethoprim-sulfamethoxazole, *TE* Tetracycline, *Amp* Ampicillin, *CRO* Ceftriaxon, *CAF* Chloramphenicol, *CN* Gentamicin, *CIP* Ciprofloxacin

Susceptibility pattern in Gram negative isolates indicates that high susceptibility rate seen in amikacin 37 (94.9%), ciprofloxacin, ceftazidamide and gentamicin each 34 (87.2%) and highly resistance seen in ampicillin 33 (84.6%), ceftriaxone 22 (56.4%) and trimethoprim/ sulfamethoxazole 17 (43.58%). Species specific antibiotic resistance in Gram negative has also indicated, *E*.*coli* were resistant to ampicillin 8 (66.7%) but relatively no resistance rates were noted to ciprofloxacin and amikacin. *K. pneumonia* was highly resistance to ampicillin 18 (94.75%), ceftriaxone 15 (78.9%) and trimethoprim/sulfamethoxazole 8 (42.1%). It was highly sensitive to ciprofloxacin and gentamicin 16 (84.2%) each (Table 4).

**Table 4:** Antimicrobial susceptibility patterns of Gram negative bacteria from blood cultures in neonatal sepsis, University of Gondar Hospital, Northwest Ethiopia, September/2015 to May/2016

Bacterial isolates	Number of resistance to antimicrobial agents (%)									
	SXT	AMC	TE	CIP	CN	CRO	AK	AMP	CAZ	CAF
*E.coli* (*n* = 12)	04 (33.3)	3 (25)	2 (16.7)	0 (0)	1 (8.3)	3 (25)	0 (0)	8 (66.7)	1 (8.3)	2 (16.7)
*K.pneumoniae* (*n* = 19)	8 (42.1)	6 (31.6)	7 (36.8)	3 (15. 8)	3 (15.8)	15 (78.9)	2 (10.52)	18 (94.7)	2 (10.5)	6 (31.6)
Others (*n* = 08)	05 (62.5)	02 (25)	01 (12.5)	02 (25)	01 (12.5)	04 (50)	0 (0)	07 (87.5)	02 (25)	02 (25)
Total = 39	17 (43.58)	11 (28.2)	10 (25.6)	5 (12.8)	5 (12.8)	22 (56.4)	2 (5.1)	33 (84.6%)	5 (12.8)	10 (25.6)

Key: *SXT* Trimethoprim- sulfamethoxazole, *AMC* Amoxicillin-clavulanate, *TE* Tetracycline, *Amp* Ampicillin, *CIP* Ciprofloxacin, *CN* Gentamicin, *AK* Amikacin, *CRO* Ceftriaxon, *CAZ*: Ceftazidamide, *CAF* Chloramphenicol, Others:*Seratia*species (01), *Enterobactor* species (04) and *K. rhinose* (03)

### Multi drug resistance pattern of bacterial isolates from neonatal septicemia

Among the total isolates, 78 (65%, 95% CI: 56.7–72.5%) were resistance to three or more different class of antibiotics. Among MDR strains, 21 (17.5%) isolate were resistant to three classes of antibiotics, the rest 57 (47.5%) were resistant to greater than three classes of antibiotics. Result of drug resistance patterns compared within species specific showed that, 33 (67.3%) of *S. aureus*, 8 (66.7%) of *E. coli* and 16 (84.2%) of *K. pnuemonae* were MDR isolates*.* Among the total isolates of *S. aureus*, 13 (26.5%) of them were found to be MRSA (Table 5).

**Table 5:** Multi drug resistance pattern of bacterial isolates in neonatal septicemia, University of Gondar Hospital North West Ethiopia, from September/2015 to May/2016

Organism isolated	Degree of resistance								Total MDR isolates ≥R3
	R0 (%)	R1 (%)	R2 (%)	R3 (%)	R4 (%)	R5 (%)	R6 (%)	R7 (%)	
*S. aureus* (*n* = 49)	08 (16.3)	02 (4)	09 (18.3)	09 (18.3)	06 (12.2)	06 (12.2)	06 (12.2)	03 (6.1)	30 (61.2)
*CoNS* *(n = 26)*	02 (7.7)	02 (7.7)	01 (3.8)	00 (0)	07 (26.9)	09 (34.6)	03 (11.5)	02 (7.7)	21 (81.0)
*S. pyogenes* *(n = 6)*	-	-	01 (16.7)	03 (50)	02 (33.3)	_	_	_	05 (83.3)
*E. coli* *(n = 12)*	-	04 (33.3)	05 (41.7)	02 (16.7)	_	_	01 (8.3)	_	03 (25.0)
*K.pnuemoniae(n = 19)*	01 (5.2)	02 (10.5)	02 (10.5)	05 (26.3)	04 (21)	01 (5.2)	02 (10.5)	02 (10.5)	14 (74.0)
Others (*n* = 8)	_	01 (12.5)	02 (25)	02 (25)	_	01 (12.5)	02 [25]	_	05 (62.5)
Total *n* = 120	11 (9.2)	11 (9.2)	20 (16.7)	21 (17.5)	19 (15.8)	17 (14.2)	14 (11.6)	07 (5.8)	78 (65.0)

Key:*CoNS Coagulase Negative Staphylococcus*, others: *Seratia*species, *E. cloaca*, *K. rehinose*, R0: susceptible to all antibiotics, R1–7: resistance to 1,2, 3, 4, 5, 6 & 7 antibiotics, ≥ R3: resistance to 3 or more antibiotic

### Associated risk factors related to neonatal septicemia

Risk factors associated with neonatal septicemia were analyzed. Univariate and multivariate analysis showed that very low birth weight with adjusted odds ratio (AOR) 12.37 (95% CI: 4.135–37.04), low birth weight with AOR 2.63 (95% CI: 1.149–6.09),caesarean section mode of delivery with AOR 5.2(95% CI: 2.36–11.37), Apgar score < 7 with AOR 0.438 (95% CI: 0.215–0.892) and preterm gestational week with AOR 8.99 (95% CI: 4.175–19.38) were associated with neonatal septicemia (Table 6).

**Table 6:** Univariate and multivariate analysis on risk factors associated with positivity rate of neonatal Septicemia at University of Gondar Hospital, Northwest Ethiopia September/2015 to May/2016

Variable	Bacterial isolates		Univariate analysis		Multi variate analysis	
	Yes = 117	No = 134	COR(95% CI)	*P*- value	AOR(95% CI)	*P*-value
Mode of delivery						
CS	52	29	2.989 (1.714,5.21)	<0.001	5.191 (2.36,11.37)	<0.001
I	5	5	1.667 (0.463,599)	0.434	5.26 (0.875,31.7)	0.070
SVD	60	100	1		1	
Gestational week						
Pre term	75	17	10.7 (5.607,20.074)	<0.001	8.99 (4.175,19.38)	<0.001
Term	42	101	1		1	
Birth weight						
Very low	40	6	17.3 (6.823,44.03)	<0.001	12.37 (4.135,37.04)	<0.001
Low	37	24	4.0 (2.135,7.526)	<0.001	2.63 (1.149,6.09)	0.022
Normal	40	104	1		1	
Apgar score						
Low	49	69	0.679 (0.412,1.12)	0.129	0.438 (0.215,0.892)	0.023
Normal	68	65	1		1	

Key: *COR* crude odds ratio, *AOR* Adjusted odds ratio, *CI* Confidence interval, *APGAR score* Appearance, pulse, grimace, activity and respiration, *CS* caesarean section, *SVD* Spontaneous Vaginal Delivery, *I* Instrumental

Multivariate analysis result indicates that gestational week has significant association which means that neonates born in gestation < 37 weeks had almost nine times more likely to develop sepsis compared to those neonates born in gestation ≥37 weeks. Neonates born with very low birth weight < 1.5 kg had 12 times more likely to develop neonatal sepsis as compared to normal birth weight > 2.5 kg. Neonates born with birth weight < 2500 Gram had 3 times more likely to develop sepsis than neonates with normal birth weight. Apgar score < 7 pre five minute had 0.5 times more likely to develop sepsis than neonates with normal Apgar score. Caesarian section mode of delivery had 5 time risk to develop neonatal sepsis than spontaneous vaginal delivery.

## Discussion

The overall culture positivity rate of bacterial isolates identified from patients with symptoms of neonatal septicemia were 46.6%, comparable with the results reported from Addis Ababa (44.7%) and Egypt (40.7%) [23, 24]. But, it is lower than study done in Sudan (61.3%) and Yemen (57%) [25, 26]. On the other hand, the present study is higher than from study done in Nepal (20.3%), Tanzania (19.2%) and Gondar (32.1%) [27–29]. This could be due to fact methodological variation, and difference in study setting might affect culture positivity rate. Besides, in previous study in Gondar, antibioti was seen that could minimize culture positivity rate.

In our finding, the predominant isolates from Gram positive and negative bacteria were *S. aureus,* 49 (40.8%) and *K. pneumoniae*, 19 (15.8%), respectively. Similarly, previous studies claimed that those isolates were also the major pathogens of neonatal septicemia [24–31]. In fact *S. aureus* is ubiquitous in nature, which is frequently found on the skin, and main cause of various type of infection in human beings, therefore, newborns can easily contaminated by this bacteria from their mothers during or after delivery [32, 33]. Likewise, *K. pneumoiae* is a normal member of gastro-intestinal flora and recently, has emerged as a significant cause of hospital acquired infections (urinary tract infection, pneumonia and septicemia). Particularly pre-mature populations are easily colonized by the bacteria, due to the fact that, infection of neonates by this bacterium is inevitable [34, 35].

Moreover, the overall MDR prevalence was 78 (65%). Among MDR Gram positive and negative bacteria, *S. aureus*, 30 (61.2%) and *K. pneumoniae,* 14 (74%) were the principal MDR strains. It is understood that *S. auerus* has remarkable feature to adapt antimicrobial pressures. Largely, it has genetic competence to acquire antibiotic resistance genes from other strains. *K. pneumonia* has also intrinsic resistance mechanisms, most importantly, it has chromosomal and plasmid encoded beta-lactam hydrolyzing enzymes (e.g. ESBLs) [36, 37]. Similarly, the issue of antibiotic resistance in the above mentioned bacteria have also documented in previous reports [28, 31, 38–40].

Specific antibiotic resistance has also indicated in the present study. Hence, ampicillin was the most ineffective antibiotics for both Gram positive and negative bacteria. It is well understood; the consequence of ampicillin resistance is mainly due to selective pressure excreted by overuse of the antibiotics. Apart from that, it is also known that bacteria showed resistance genes for beta-lactam agents including ampicllin. Similarly, consistent finding were also claimed in Addis Ababa, Gondar and Egypt with high degree of resistance to ampicillin in both Gram positive and negative bacteria [23, 28, 38, 39].

In our study, the incidence of sepsis was higher in neonates, who were born by caesarian section, thus the odds of developing sepsis among neonates born with caesarian section was five times more likely to develop sepsis to their counterparts. This finding is similar to other previous studies from Iran and Egypt [23, 38]. Reasonably, bleeding is common during surgical procedures, thus, it could be the possible factor that results to blood contamination, subsequently leads bacterial sepsis in neonates. Particularly, Neonates are vulnerable to nosocomial infections because of their reduced immunological profiles as well as the invasive procedures to which they are subjected. This is highly appreciated for those born prematurely or of low birth weight [41]. Likewise, different studies came up with the conclusion that low birth weight infants are at high risk of developing sepsis compared to normal birth weight [42].

It is largely understood that prolonged rupture of membrane and prolonged labor are the commonly associated risk factors for the occurrence of neonatal sepsis because of the danger of ascending infection. However, the present study indicated that no significant associations with the above mentioned variables. This study showed that low Apgar score at 5th minute had a significant effect on the development of neonatal sepsis; this result is consistence with study conducted in Mekelle, Indonesia and Nepal [43–45]. In general Apgar score at the first minute associated with the Hydrogen Potential (pH) cord blood and intra partum depression and not associates with the outcomes, whereas the Apgar score/5 min then reflects the infants’ changes condition on the resuscitation performed [44].

In the present study, gestational week <37 was also the risk factor with nine times higher risk to develop sepsis than gestational week >37. Similarly, studies conducted in Addis Ababa, Nepal, Mexico and Indonesia, have also documented that gestation <37 weeks had significant association with neonatal sepsis [39, 43, 44, 46]. Evidently, preterm baby may have a limited capacity to increase neutrophil production in accordance to demand to overcome the problem. Neutropenia and depleted neutrophil storage pools have been found to be associated with neonatal bacterial sepsis and are predictive of a poor prognosis [47].

In this study very low birth weight and low birth weight had significant association with the occurrence of sepsis; this is consistent with studies conducted in Nepal and Indonesia [43, 44]. Immunological barriers began to mature around 32–34 weeks of gestation and accelerated after birth. That’s why the level mucosal antibody is low in underweight neonates [48]. the deficiency of molecular reactants phase such as C- reactive proteins, inhibitor protein and several of the coagulation proteins have been also demonstrated in immature neonates. Moreover, pre-term baby had a lot of neutrophil in circulatory pool but it reserves in the bone marrow is only about 20% compared to the term baby and adults so that the state of sepsis occur severe neutropenia [48–50].

### Limitation of the study

A specific characterization MDR bacterium (MRSA, ESBL) has not been done due to lack of well-established methodological techniques. Moreover, molecular based specification for some bacterial groups (CoNS) was not done.

## Conclusion

High prevalence of bacterial isolates was observed in this study. *S. aureus* was the predominant pathogen from Gram positive and *K. pneumonia* was from Gram negative. The overall prevalence of MDR was high. Comparably high level of resistance to ampicillin was observed among Gram positive and Gram negative bacteria.The most common risk factors for neonatal sepsis infection observed in this study were caesarian section mode of delivery, Apgar score < 7, birth weight < 2.5 kg and gestational week <37 weeks. Substantially, strengthening of antenatal screening of mothers, prenatal care of newborns and interventions of babies born with complications are the key elements to control the problem. Moreover, Empirical regimens for neonatal sepsis using ampicillin and gentamicin must be taken into consideration due to the risk of resistance, misdiagnosis and mismanagement.

## Additional file

Additional file 1: Questionnaire. (DOCX 14 kb)

## Abbreviations

CoNS: Coagulase negative *Staphylococcus* species
CSF: Cerebrospinal fluid
ESBL: Extended spectrum beta lactamase
MDR: Multi Drug resistant
MRSA: Methicilin resistant *staphylococcus aureus*
UTI: Urinary tract infection
