# Supplementary material for: Bacterial etiologic agents causing neonatal sepsis and associated risk factors in Gondar, Northwest Ethiopia
Source: BMC Pediatr. 2017 Jun 6;17:137. doi: 10.1186/s12887-017-0892-y (PMC5461759; doi:10.1186/s12887-017-0892-y)
Supplement: Additional file 1: — Questionnaire. (DOCX 14 kb) [file 12887_2017_892_MOESM1_ESM.docx]

## Additional file 1: Questionnaire

Data collection form for the Bacterial etiologic agent that causes neonatal sepsis, their susceptibility pattern and associated risk factor at University of Gondar Hospital, North-West Ethiopia.

Patient ID -------------- Hospital ward……… Date ……….

Section 1: Socio demographic information:

| S/no | Question | Code | Response |
| --- | --- | --- | --- |
| 101 | Age | **-----**hrs(if<72hours)  ------days(if >72 hours) |  |
| 102 | Sex | 0=Male  1=Female |  |
| 103 | Residence | 0=Urban  1=Rural |  |

**Section 2: Risk factor assessment**

**2.1: Mother conditions**

| S/no | Question | Code | Response |
| --- | --- | --- | --- |
| 201 | Did you have prolonged labor greater than 24 hrs? | 0= No  1= Yes |  |
| 202 | Did you have prolonged rupture of membrane greater than 18-24 hours(between time of rupture and time of delivery) | 0= NO  1= Yes |  |
| 203 | Did you have fever during pre partum period | 0= N0  1= Yes |  |
| 204 | Mode of delivery | 0= caesarian section  1= Spontaneous vaginal delivery  2=Instrumental |  |
| 205 | Place of delivery | 0=Home  1=Health facility  2=Health post  3=On transport |  |
| 206 | Did you have UTI during pregnancy | 0= N0  1= Yes |  |
| 207 | If your answer to number 206 Q yes did you get treatment by health personnel | 0= No  1= Yes |  |

Section2.2: Neonate condition

| S. no | Question | Code | Response |
| --- | --- | --- | --- |
| 301 | After how many days of delivery your neonate become sick | 1= <3 days  2= ≥ 3 days |  |
| 302 | At what gestational age you gave birth | 1. 37-42 weeks  2. <37 week  3. > 42 weeks |  |
| 303 | Birth weight | 1. ≥2500 g  2. <1500 g  3. 1500-2500 g |  |
| 304 | APGAR score | 1.>7/5minute  2.<7/5minute |  |
| 305 | Previous history of neonatal admission | 1. No  2. Yes |  |
| 306 | Bottle feeding | 1. No  2. Yes |  |
| 307 | Is your neonate having skin /local infection? | 1. No  2. Yes |  |
